# Rigid Nanoporous Urea-Based Covalent Triazine Frameworks for C2/C1 and CO_2_/CH_4_ Gas Separation

**DOI:** 10.3390/molecules26123670

**Published:** 2021-06-16

**Authors:** Chidharth Krishnaraj, Himanshu Sekhar Jena, Florence Lecoeuvre, Karen Leus, Pascal Van Der Voort

**Affiliations:** COMOC, Center for Ordered Materials, Organometallics and Catalysis, Department of Chemistry, Ghent University, 9000 Gent, Belgium; chidharth.krishnaraj@ugent.be (C.K.); florencelecoeuvre@gmail.com (F.L.); Karen.Leus@ugent.be (K.L.)

**Keywords:** covalent triazine frameworks, covalent organic frameworks, gas separation, C2/C1 hydrocarbon separation, C_2_H_2_/CH_4_, CO_2_/CH_4_

## Abstract

C2/C1 hydrocarbon separation is an important industrial process that relies on energy-intensive cryogenic distillation methods. The use of porous adsorbents to selectively separate these gases is a viable alternative. Highly stable covalent triazine frameworks (urea-CTFs) have been synthesized using 1,3-bis(4-cyanophenyl)urea. Urea-CTFs exhibited gas uptakes of C_2_H_2_ (3.86 mmol/g) and C_2_H_4_ (2.92 mmol/g) at 273 K and 1 bar and is selective over CH_4_. Breakthrough simulations show the potential of urea-CTFs for C2/C1 separation.

## 1. Introduction

The separation of C1 and C2 gases is a critical process in many industrial activities. For example, acetylene is an important industrial byproduct of petroleum and natural gas processing, which needs to be separated. In addition, there are other industrial processes wherein ethylene and acetylene are produced by the oxidative and non-oxidative coupling of methane [1]. However, quite often the methane conversion remains incomplete and recovering the unreacted methane is essential [2]. As another example, in the process of extracting natural gas, methane needs to be separated from carbon dioxide [3]. Natural gas consists of high amounts of carbon dioxide that must be removed to obtain pure methane, which can be used as an energy source for fuels and chemicals. As a final example, the separation of CO_2_ in flue gases (typically containing about 75% nitrogen and traces of water (vapor)) is becoming an important process in carbon capture and utilization CCU strategies.

Metal organic frameworks (MOFs) have been studied for this purpose [4,5,6]. Although some show very high adsorption capacities and selectivities, they often lack long-term stability, an important factor for a potential adsorbent [7]. Hence, other types of porous adsorbents, such as porous organic polymers are also considered for such gas separation processes.

Covalent triazine frameworks (CTFs) are a class of organic porous materials that can be used for gas separation [8,9,10]. Research on CTFs has boomed due to their ease of synthesis, tunable porosities, desirable functionalization, and ultra-high stability [11,12,13,14]. They are primarily made through an ionothermal synthesis, where ZnCl_2_ is used as an ionic liquid solvent and catalyst for the trimerization of dinitrile linkers. The ionothermal synthesis method has so far been used to design several inherent functionalities in CTFs, such as fluorine containing CTF (FCTF-S, F-DCBP-CTF) [15,16], acetylacetone containing CTF (acac-CTF) [17], bipyridine containing CTF (bpy-CTF) [18], ionic CTF (cCTF) [19], porphyrin CTFs [20], N-heterocyclic carbene CTF (NHC-CTF) [21], binol [22], etc. The produced materials show good properties for several applications in gas storage and separation [23], catalysis [24], electrocatalysis [25,26], photocatalysis [27,28], and batteries [29].

**Scheme 1:**
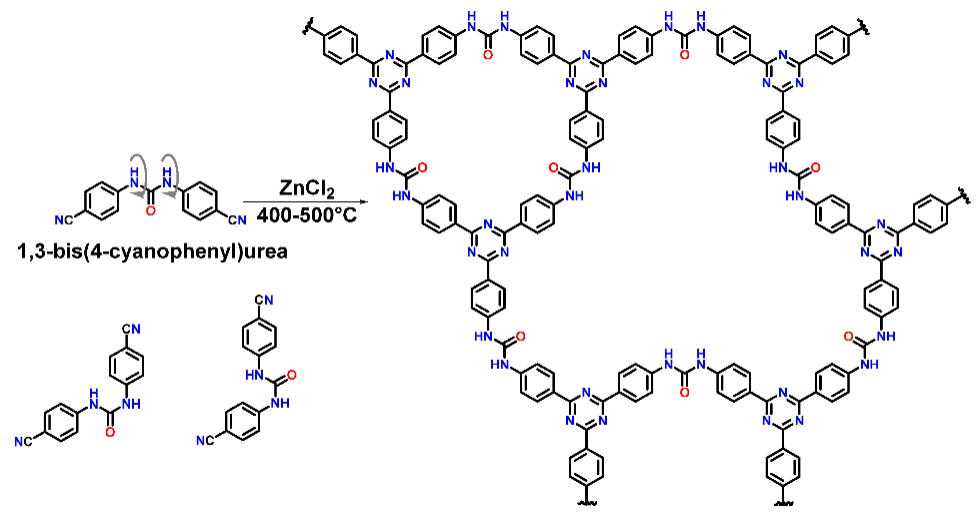
Schematic representation of the synthesis and ideal structure of the urea-functionalized CTFs. 1,3-bis(4-cyanophenyl)urea is a flexible linker, and its possible conformations are listed.

However, these harsh ionothermal synthesis conditions result in materials that are mostly amorphous. In some reports, CTFs with a partial crystallinity were obtained [10,12]. Due to carbonization at these high temperatures, the structural characterization of the material becomes arduous. Nonetheless, CTFs exhibit exceptional properties in comparison to other covalent organic framework (COFs) for the above noted applications. One of the most appealing properties of the ionothermally synthesized CTFs is their exceptionally high thermal, hydrothermal, and chemical stability. They can withstand temperatures up to 550 °C and extreme chemical environments, such as 1 M NaOH or 1 M HCl solutions, for an extended period. Such stability is important for “real life” gas adsorption/separation applications, wherein high temperatures and the acid/base poisoning of the gas streams are important considerations [30]. This encouraged us to design new CTFs, particularly with polar functional sites that can be beneficial for gas storage and separations. Recently, Yaghi et al., reported the first urea-linked ketoenamine COFs and highlighted their structural dynamics with respect to their flexibility [31]. However, the COFs were not highly stable in basic conditions (1 M NaOH). In order to develop porous materials that are stable under both strong acidic and basic conditions, we report herein the synthesis of ultra-stable urea-based CTFs using a dinitrile linker, (1,3-bis(4-cyanophenyl)urea) (Scheme 1). We studied their surface properties as well as their potential for C_2_H_2_, C_2_H_4_, and CO_2_ separation over CH_4_. We report herein that urea-CTFs display high C_2_H_2_ and C_2_H_4_ uptakes and moderate CO_2_ adsorption capacity in comparison to the existing CTFs. Moreover, the C2 hydrocarbon (C_2_H_2_ and C_2_H_4_) adsorption was selective compared to C1 hydrocarbon (CH_4_). In addition, urea-CTFs also exhibited good selectivity for CO_2_ over CH_4_.

## 2. Results and Discussion

### 2.1. Synthesis and Characterization of Urea-CTFs

For the synthesis of the targeted urea-based CTFs, the linker 1,3-bis(4-cyanophenyl)urea was synthesized from 4-aminobenzonitrile according to the reported procedure [32]. In general, urea-CTFs were obtained through ionothermal synthesis using ZnCl2 (5 eq.) both as a catalyst and a solvent at 400 °C (urea-CTF-400-5) and 500 °C (urea-CTF-500-5) (Scheme 1, ESI). The complete trimerization of the cyano (-CN) groups was confirmed through Fourier transform infrared (FTIR) analysis (Figure S1) where the -CN peak at 2226 cm^−1^ of the monomer is no longer visible in the CTFs [10,14]. In addition, triazine peaks were observed around 1360 cm^−1^ and 1600 cm^−1^, which further confirm the successful trimerization. Notably, a small broad peak around 1707 cm^−1^ was observed in the CTFs that are red-shifted from 1737 cm^−1^ of C(O) monomer and confirms the presence of urea groups in the resulting materials [31]. The observed lower wavenumber might be due to the decrease in the double-bond character of the C(O) bond of the urea functional group after the CTF formation.

**Figure 1:**
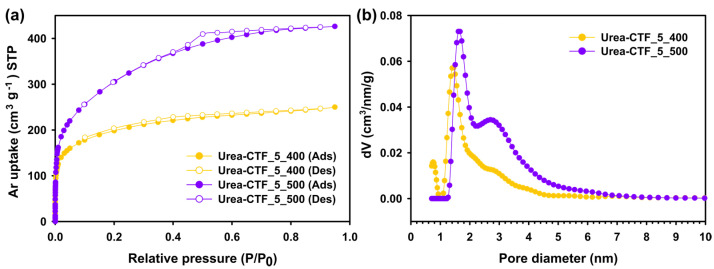
(**a**) Argon sorption isotherms measured at 87 K and (**b**) the pore-size distribution of the urea-CTFs based on quenched solid density functional theory (QSDFT) cylindrical pores.

The porous properties of both the CTF materials were explored using argon sorption at 87 K (Figure 1) and N_2_ sorption measurements at 77 K (Figure S2). Both urea-CTF_400_5 and urea-CTF_500_5 displayed a Type I isotherm typical for microporous materials, and the calculated BET surface areas were 555 m^2^ g^−1^ and 928 m^2^ g^−1^, respectively. The detailed textural properties are described in Table S1. As seen in several reported CTFs [11], the microporosity content depends on the synthesis temperature, whereas, urea-CTF_400_5 shows a higher microporous-to-mesoporous volume ratio in comparison to Urea-CTF_500_5. The theoretical expected pore sizes are 0.7 nm and 1.4–1.5 nm as shown in the structure (Scheme S1). From the experimental argon pore-size distribution, 0.75/1.43 nm pores for urea-CTF_400_5 and 1.65/2.70 nm pores for urea-CTF_500_5 were obtained. The values for urea-CTF_400_5 correspond well with the expected pore size, whereas, for urea-CTF-500, the absence of the smallest pore (0.7 nm) and the appearance of a larger pore (2.70 nm) were observed. This is the result of thermal decomposition causing the fragmentation of the walls on top of the micropores, creating mesopores in urea-CTF_500_5 [33]. A higher synthesis temperature also causes a higher degree of carbonization [34], which is seen in the C/N ratio from the elemental analysis data. The presence of a sudden drop in the adsorbed volume in the desorption isotherm at P/P_0_~0.45 (Figure S2) is due to the tensile strength effect leading to a forced closure of the hysteresis loop [35]. The powder X-ray diffraction (PXRD) analysis show the amorphous characteristics of the materials with a broad diffraction band at 2θ = 25.8 degrees (Figure S3). The physicochemical stability of the urea-CTFs was analyzed using thermogravimetric analysis (TGA) which showed that the materials were stable up to 450 °C (Figure S4). In addition, the chemical stability of the urea-CTF_400_5 and urea-CTF_500_5 material was studied by exposing them to boiling water (3 days), 6 M NaOH (3 days), and 6 M HCl (3 days). After each treatment, they were cleaned to remove the corresponding chemical traces, and N_2_ sorption was performed (Figures S5 and S6). In all cases, microporosity was retained, proving the permanent microporosity of the urea-CTF. Transmission electron microscopy (TEM) images show the two-dimensional stacking of the urea-CTFs (Figures S7 and S8). In addition, scanning electron microscopy (SEM) images show that urea_CTF_400_5 particles, are on average, larger than the urea_CTF_500_5 particles (Figures S7 and S8). Lower temperature synthesis of the CTF created fewer defects, and hence, urea_CTF_400_5 had longer sheet morphology.

### 2.2. Gas Storage and Separation

**Figure 2:**
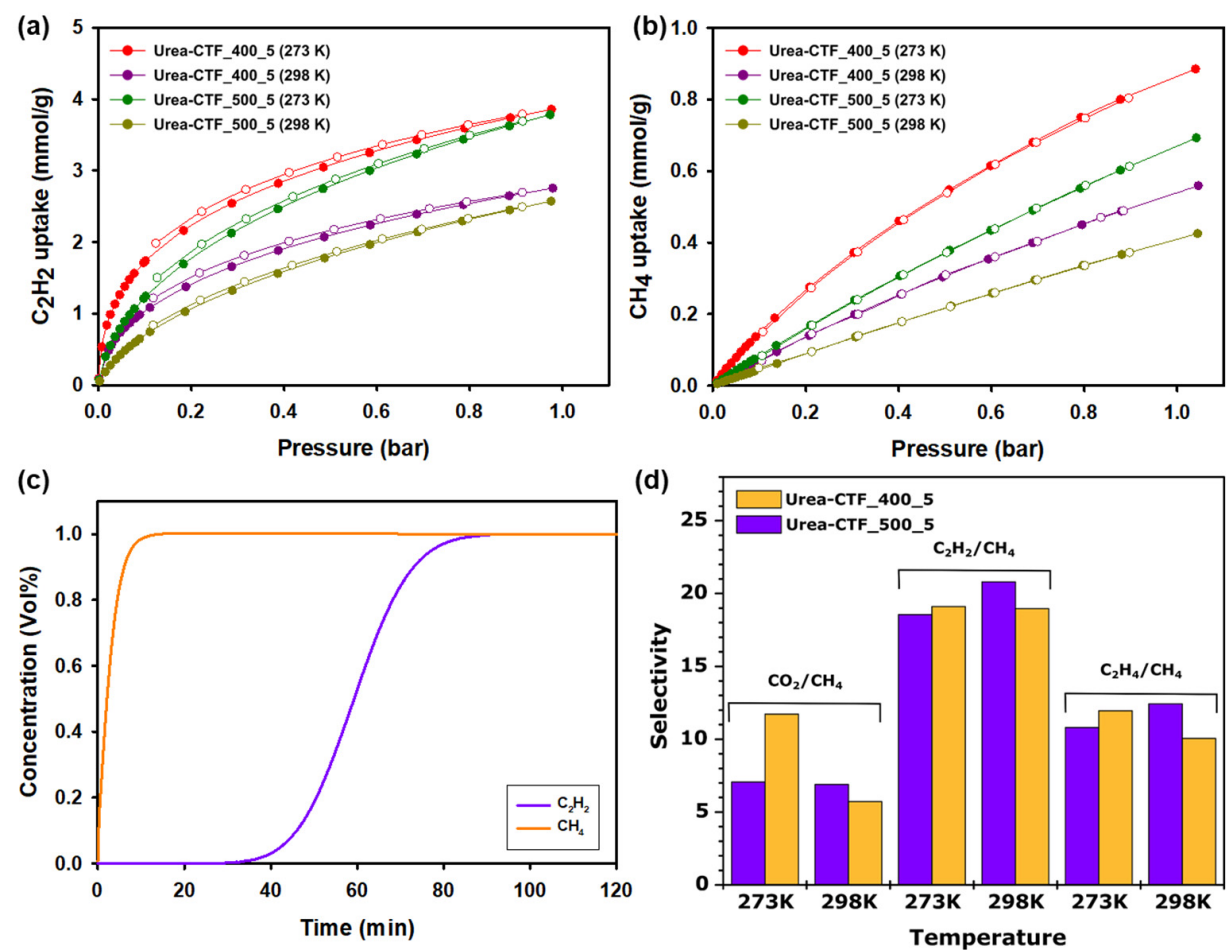
(**a**) C_2_H_2_ uptake, (**b**) CH_4_ uptake, (**c**) C_2_H_4_/CH_4_ breakthrough simulation of urea-CTF_400_5, and (**d**) CO_2_, C_2_H_2_, and C_2_H_4_ selectivities over CH_4_ of urea-CTF_400_5 and urea-CTF_500_5.

Although CTFs in general have high potential for gas storage and separation, their potential for C2 hydrocarbon storage and separation has only rarely been explored. Only recently, CTF-PO71 [36] and hexene-CTF [37] have been studied for C2 hydrocarbon storage and separation. The permanent microporosity and presence of urea/triazine functional groups make urea-CTFs excellent candidates for this purpose. To this end, C2 hydrocarbon storage capacity was tested for both urea-CTF_400_5 and urea-CTF_500_5. Among these samples, urea-CTF_400_5 showed the highest C_2_H_2_ uptake (3.86 mmol/g) at 273 K and 1 bar pressure, which is higher than the previously reported CTFs (Figure 2a).

Interestingly, despite the higher surface area of urea-CTF_500_5, a similar C_2_H_2_ uptake (3.78 mmol/g) at 273 K and 1 bar pressure was observed (Figure 2a, Table S2). This is due to the abundance of the micropores in both materials. However, for urea-CTF_400_5, the V_micro_/V_tot_ (0.72) is slightly higher than for urea-CTF_500_5 (0.61) (Table S1). This results in a steeper increase of C_2_H_2_ uptake at the lower-pressure regime for urea-CTF_400_5. In addition, similar trends were observed in C_2_H_4_ uptake (2.89 mmol/g and 2.92 mmol/g for urea-CTF_400_5 and urea-CTF_500_5, respectively) (Figure S9). The affinity at 273 K and 298 K of the C2 hydrocarbons for the urea-CTFs was calculated by the Clausius–Clapeyron equation (Figures S11 and S12). The isosteric heat of adsorption (*Q*_st_) values are given in Table S3. As expected, in both cases, a higher affinity was observed in urea-CTF_400_5 because the lower synthesis temperature resulted in fewer defects. In addition to the storage capacity, selectivity is perhaps an even more important parameter for industrial utilization. First, we targeted C_2_H_2_/CH_4_ and C_2_H_4_/CH_4_ separation. The CH_4_ uptake isotherms at 273 K and 298 K are given in Figure 2b. Selectivity was estimated using the ideal adsorbed solution theory (IAST) (Table S4 and Figures S14–S17). The calculated selectivities of the urea-CTFs were within 16.9–20.2 and 8.9–12.4 for C_2_H_2_/CH_4_ and C_2_H_4_/CH_4_, respectively, which are promising results for C2/C1 hydrocarbon separation (Figure 2d, Table 1).

The presence of inherent triazine and urea functionalities in urea-CTFs also encouraged us to test CO_2_ adsorption performance. The CO_2_ adsorption and desorption isotherms were measured at 273 K and 298 K up to 1 bar. At 1 bar and 273 K, urea-CTF_400_5 and urea-CTF_500_5 showed 2.8 mmol/g and 3.1 mmol/g uptake respectively, which are moderate values in comparison to other CTFs (Figure S10, Table S2). The heat of liquefaction of bulk CO_2_ is 17 kJ/mol [38], and urea-CTF_500_5 shows an isosteric heat of adsorption of 48.57 kJ/mol (Figure S12 and Table S3), which is much higher. In addition, the *Q*_st_ values of urea-CTFs are much higher than several reported CTFs and higher than activated carbon at low CO_2_ pressure (17.8 kJ/mol). This confirms the strong dipolar interactions between CO_2_ and the N-basic sites, as well as the H-bonding interactions between the urea functional group and the CO_2_ molecules. The selectivity of CO_2_ over N_2_ and CH_4_ are also important factors for CCS applications. CO_2_/CH_4_ and CO_2_/N_2_ selectivity were calculated using IAST (Table S4), and the best values, 20.3 and 69.6, were respectively obtained for urea-CTF_400_5 at 273 K. Notably, the obtained CO_2_/N_2_ selectivity of urea-CTF_400_5 is higher than several reported CTFs [14,39,40,41].

**Table 1:** Comparison of urea-CTFs with other materials for C_2_H_2_/CH_4_ and CO_2_/CH_4_ selectivities and adsorption enthalpies.

Material	Temp. (K)	C_2_H_2_ Uptake (mmol/g)	C_2_H_2_/CH_4_ Selectivity (273 K)	C_2_H_2_ Adsorption Enthalpy (kJ/mol)	CO_2_ Uptake (mmol/g)	CO_2_/CH_4_ Selectivity (273 K)	CO_2_ Adsorption Enthalpy	Ref.
UTSA-50	296	3.80	68	39.4	2.63	5	27.8	[4]
Zn_4_(OH)_2_(1,2,4-*btc*)_2_	295	2.22	14.7	28.2	1.72	4.5	20.2	[5]
ZJU-60a	296	6.33	-	17.6	2.99	5–5.6	15.2	[6]
Hexene-CTF_400_1	298	2.28	12.8	47	2.66	8	32	[37]
ZJU-61a	298	5.88	115.3	23.98	-	-	-	[42]
HOF-BTB	295	2.87	7.8	24.3	-	-	-	[43]
UTSA-36a	295	2.45 ^Ξ^	16.1	29.0	-	-	-	[44]
Activated carbon	303	-	-	-	3.45	2.5 (303 K)	24.2	[45]
Urea-CTF_400_5	298	2.80	20.25	35.51	1.8	10.49	30.05	This work
Urea-CTF_500_5	298	2.57	18.96	27.78	1.5	10.47	48.57	This work

Note: ^Ξ^ Extrapolated from plot.

To verify the performance of the adsorbents in a mixed component system, breakthrough simulations were performed. Urea-CTF_400_5 was selected for these simulations as it showed the best performance among the urea-CTFs in all gas separations. The affinity constants and maximal loadings at the corresponding temperatures were obtained from Langmuir adsorption isotherm fitting (ESI). With these values, the equilibrium data for a mixed-component system were simulated. The equilibrium plots for C_2_H_2_/CH_4_, C_2_H_4_/CH_4_, and CO_2_/CH_4_ components with (i) constant gas composition and variable pressure and (ii) constant pressure and variable gas composition are shown in Figures S18–S20. As expected, even in a 50:50 mixtures, uptake is higher for C_2_H_2_, C_2_H_4_, and CO_2_ as compared to CH_4_ due to the higher affinity constants. Further breakthrough simulations were performed with defined height, diameter of the column, gas-flow rate, and mass of the adsorbent at 25 °C and 1 bar pressure. The breakthrough plots for C_2_H_2_/CH_4_, C_2_H_4_/CH_4_, and CO_2_/CH_4_ are shown in Figure 2c and Figures S21 and S22. The results show promising C2/C1 and CO_2_/CH_4_ separation using urea-CTF-5-400.

## 3. Conclusions

In conclusion, rigid and highly stable CTFs were synthesized using flexible urea-based linkers. These materials exhibit high surface areas with good C_2_H_2_, C_2_H_4_, and CO_2_ adsorption properties. The calculated C_2_H_2_/CH_4_, C_2_H_4_/CH_4_, and CO_2_/CH_4_ selectivity values demonstrate that these materials are promising for C2/C1 hydrocarbon separation, as well as for the separation of CO_2_ in natural gas extraction.

## Data Availability

Not applicable.

## Supplementary Materials

The following are available online. Instrumentation, Synthesis of Urea-CTFs, FT-IR spectra, N_2_ sorption, Porous properties of Urea-CTFs, PXRD pattern, TGA spectra, Stability tests, TEM and SEM images, Gas uptake values, Isosteric heat of adsorption, IAST selectivities, Breakthrough simulations. Figure S1: FT-IR spectral comparison between Urea-CTFs obtained at different temperatures with respect to the monomer, Figure S2: N_2_ sorption isotherms of the Urea-CTFs, Figure S3: PXRD pattern of the obtained Urea-CTFs, Figure S4: TGA spectra of the obtained Urea-CTFs, Figure S5: N_2_ sorption isotherms of (i) Urea-CTF_400_5, (ii) Urea-CTF_400_5 in boiling water for 3 days, (iii) Urea-CTF_400_5 in 6M NaOH for 3 days, and (iv) Urea-CTF_400_5 in 6M HCl for 3 days, Figure S6: N_2_ sorption isotherms of (i) Urea-CTF_500_5, (ii) Urea-CTF_500_5 in boiling water for 3 days, (iii) Urea-CTF_500_5 in 6 M NaOH for 3 days, and (iv) Urea-CTF_500_5 in 6 M HCl for 3 days, Figure S7: TEM and SEM images of Urea-CTF_400_5, Figure S8: TEM and SEM images of Urea-CTF_500_5, Figure S9: C_2_H_4_ uptake of Urea-CTF_400_5 and Urea-CTF_500_5. Figure S10: CO_2_ uptake of Urea-CTF_400_5 and Urea-CTF_500_5, Figure S11: Isosteric heat of adsorption (C_2_H_2_, C_2_H_4_, CO_2_, CH_4_, N_2_) for the Urea-CTF_400_5, Figure S12: Isosteric heat of adsorption (C_2_H_2_, C_2_H_4_, CO_2_, CH_4_) for the Urea-CTF_500_5, Figure S13: N_2_ uptake of Urea-CTF_400_5 and Urea-CTF_500_5, Figure S14: IAST selectivity of Urea-CTF_500_5 at 273 K, Figure S15: IAST selectivity of Urea-CTF_500_5 at 298 K, Figure S16: IAST selectivity of Urea-CTF_400_5 at 273 K, Figure S17: IAST selectivity of Urea-CTF_400_5 at 298 K, Figure S18: C_2_H_2_ vs CH_4_ (mixed component simulation) at (left) constant gas composition and variable pressure and (right) variable gas composition and constant pressure, Figure S19: C_2_H_4_ vs CH_4_ (mixed component simulation) at (left) constant gas composition and variable pressure and (right) variable gas composition and constant pressure, Figure S20: CO_2_ vs CH_4_ (mixed component simulation) at (left) constant gas composition and variable pressure and (right) variable gas composition and constant pressure, Figure S21: Breakthrough simulation of C_2_H_4_ vs CH_4_ for Urea-CTF_400_5, Figure S22: Breakthrough simulation of CO_2_ vs CH_4_ for Urea-CTF_400_5, Scheme S1: Theoretical pore sizes in Urea-CTF, Table S1. Surface area and pore volume based on Argon sorption at 87K and elemental content of Urea- CTF, Table S2: Gas uptake values for the Urea-CTFs at 1 bar pressure, Table S3. Isosteric heat of adsorption (Qst) values for the Urea-CTFs, Table S4: IAST selectivity values of the Urea-CTFs, Table S5: Langmuir fit parameters for Urea-CTFs, Table S6: Fitting parameters of adsorption isotherms of Urea-CTF_400_5 at 298 K, Table S7: Breakthrough simulation parameters for Urea-CTF_400_5 at 298 K.

## Sample Availability

Samples of the Urea-CTFs are available from the authors.
