# Supplementary material for: Rigid Nanoporous Urea-Based Covalent Triazine Frameworks for C2/C1 and CO2/CH4 Gas Separation
Source: Molecules. 2021 Jun 16;26(12):3670. doi: 10.3390/molecules26123670 (PMC8235060; doi:10.3390/molecules26123670)
Supplement: Supplementary file 1 [file molecules-26-03670-s001.zip › molecules-1248039-supplementary.pdf]

Electronic Supporting Information

Rigid Nanoporous Urea-based Covalent Triazine Frameworks for C2/C1 and CO_2/CH_4 Gas Separation

Chidharth Krishnaraj [1,†], Himanshu Sekhar Jena [1,†], Florence Lecoeuvre [1], Karen Leus [1], and Pascal Van Der Voort [1,*]

[1] COMOC, Center for Ordered Materials, Organometallics and Catalysis, Department of Chemistry, Ghent University, 9000 – Gent, Belgium
* Correspondence: PVDV - pascal.vandervoort@ugent.be

KEYWORDS Gas Separation, Covalent Triazine Frameworks, Porous Materials, Methane Separation, Adsorption

I. Instrumentation:

The chemicals were purchased from Sigma-Aldrich and used without further purifications unless mentioned otherwise. A Thermo Scientific Flash 2000 CHNS-O analyzer equipped with a TCD detector was used to perform elemental analysis (C/H/N). Nitrogen sorption isotherms were measured on a Belsorp Mini apparatus at 77K. A Thermo Nicolet 6700 FTIR spectrometer equipped with a nitrogen cooled MCT detector and a KBr beam splitter for the region of 4000-650 cm^{-1} was used to perform the Fourier transform infrared spectroscopy (FT-IR). X-Ray powder diffraction (XRPD) measurements were done on a Thermo Scientific ARL X'Tra diffractometer, operated at 40 kV, 30 mA using Cu Kα radiation (λ = 1.5406 Å). Carbon dioxide (CO_2), Ethlyene (C_2H_4), Acetylene (C_2H_2), Methane (CH_4) and Nitrogen (N_2) gas adsorption isotherms were collected using a Quantachrome iSorb-HP gas sorption analyzer. Thermogravimetric analysis (TGA) was performed on a Netzsch STA-449 F3 Jupiter-simultaneous TG-DSC analyzer within a temperature range of 20-800°C, under a N_2 atmosphere and heating rate of 2°C/min. Argon sorption was performed on Quantachrome AutosorbiQ at 87K.

II. Experimental:

Synthesis of Urea-CTFs. In general, 1,3-bis(4-cyanophenyl)urea (263 mg, 1 mmol) was charged in an ampoule with 5 equivalents of $ZnCl_2$ in a glovebox. Hereafter, the ampoule was evacuated, flame-sealed and slowly heated to the desired temperature (400°C /500°C) with a heating rate of 1°C/min for 48 hours in a Nabertherm furnace oven. The products were obtained as monoliths which were crushed and cleaned with distilled water, 1 M HCl (reflux) and tetrahydrofuran (THF). The final product was activated at 150°C under vacuum for 12 hours.

III. Results:

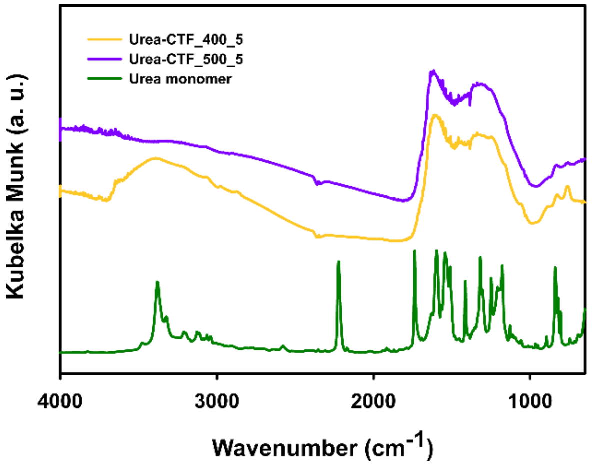

Figure S1: FT-IR spectral comparison between Urea-CTFs obtained at different temperatures with respect to the monomer.

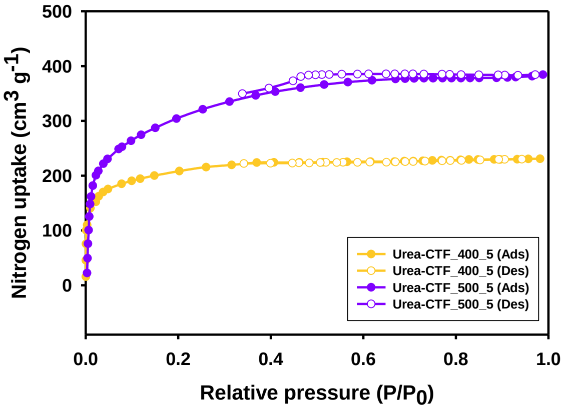

Figure S2: N_2 sorption isotherms of the Urea-CTFs.

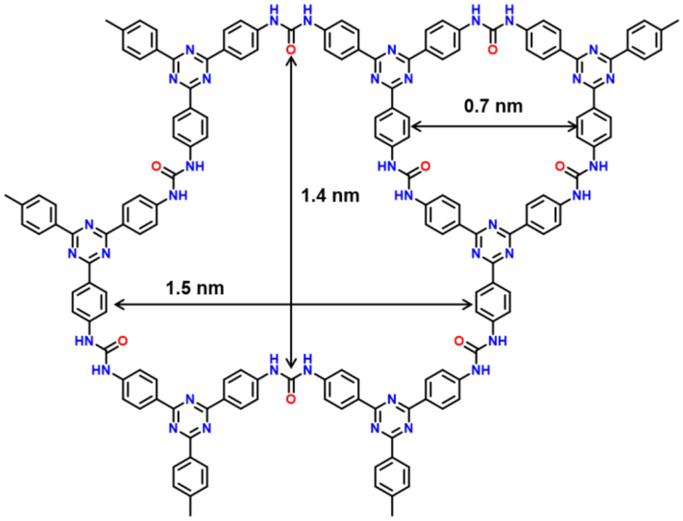

Scheme S1: Theoretical pore sizes in Urea-CTF.

Table S1: Surface area and pore volume based on Argon sorption at 87K and elemental content of Urea-CTF.

Material	Surface area ($m^2 g^{-1}$) BET	V_{tot} (cm^3g^{-1})	V_{micro} (cm^3g^{-1})	V_{micro}/V_{tot}	C (%)	H (%)	N (%)	C/N ratio	Excess carbon content (carbonization %)
Urea-CTF_400_5	555 (551*)	0.314	0.226	0.72	65.65	2.09	14.00	4.69	8.77
Urea-CTF_500_5	928 (982*)	0.533	0.325	0.61	64.86	1.93	11.38	5.70	14.28
Theory	-	-	-		68.69	3.84	21.36	3.22	--

*represents the surface area calculated using N_2 sorption isotherms

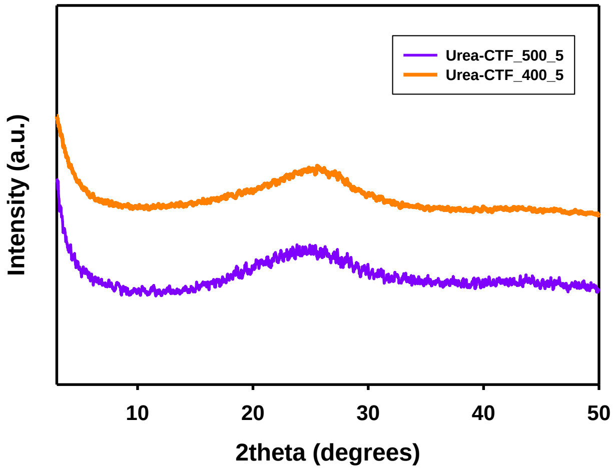

Figure S3: PXRD pattern of the obtained Urea-CTFs.

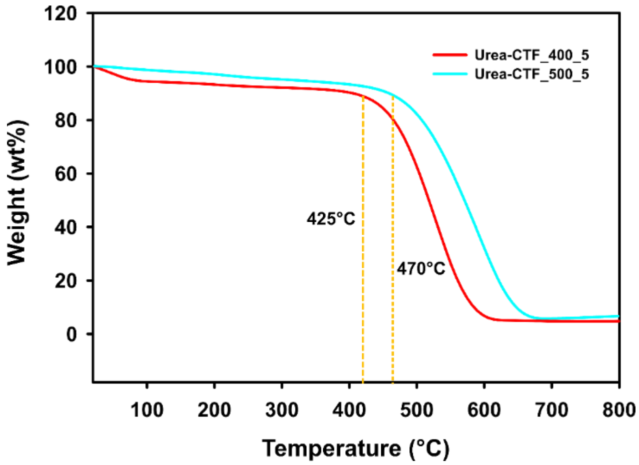

Figure S4: TGA spectra of the obtained Urea-CTFs.

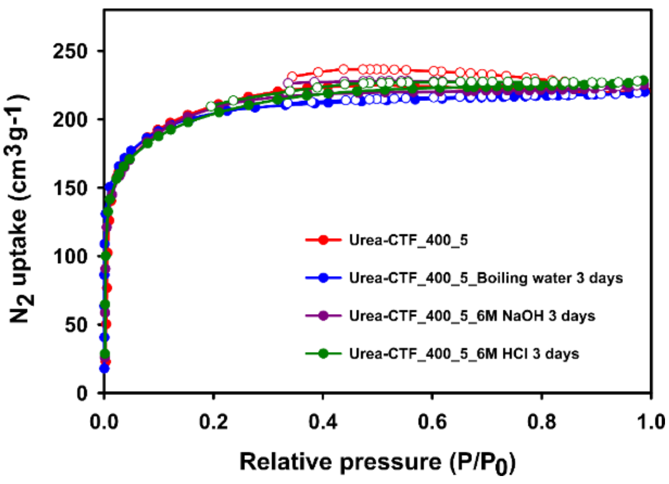

Figure S5: N_2 sorption isotherms of (i) Urea-CTF_400_5, (ii) Urea-CTF_400_5 in boiling water for 3 days, (iii) Urea-CTF_400_5 in 6M NaOH for 3 days, and (iv) Urea-CTF_400_5 in 6M HCl for 3 days.

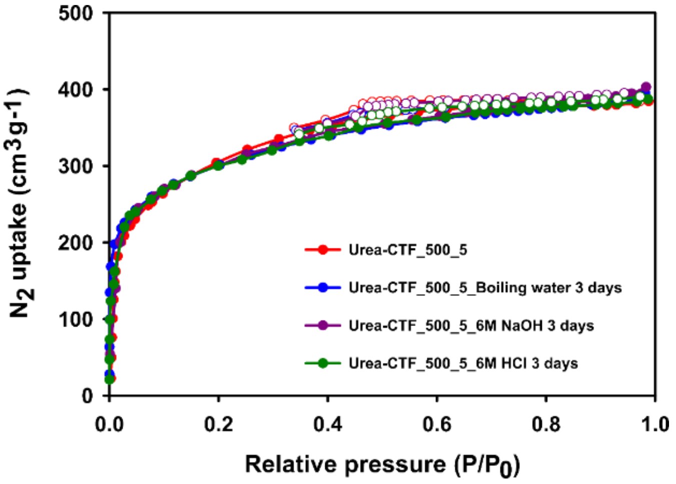

Figure S6: N_2 sorption isotherms of (i) Urea-CTF_500_5, (ii) Urea-CTF_500_5 in boiling water for 3 days, (iii) Urea-CTF_500_5 in 6M NaOH for 3 days, and (iv) Urea-CTF_500_5 in 6M HCl for 3 days.

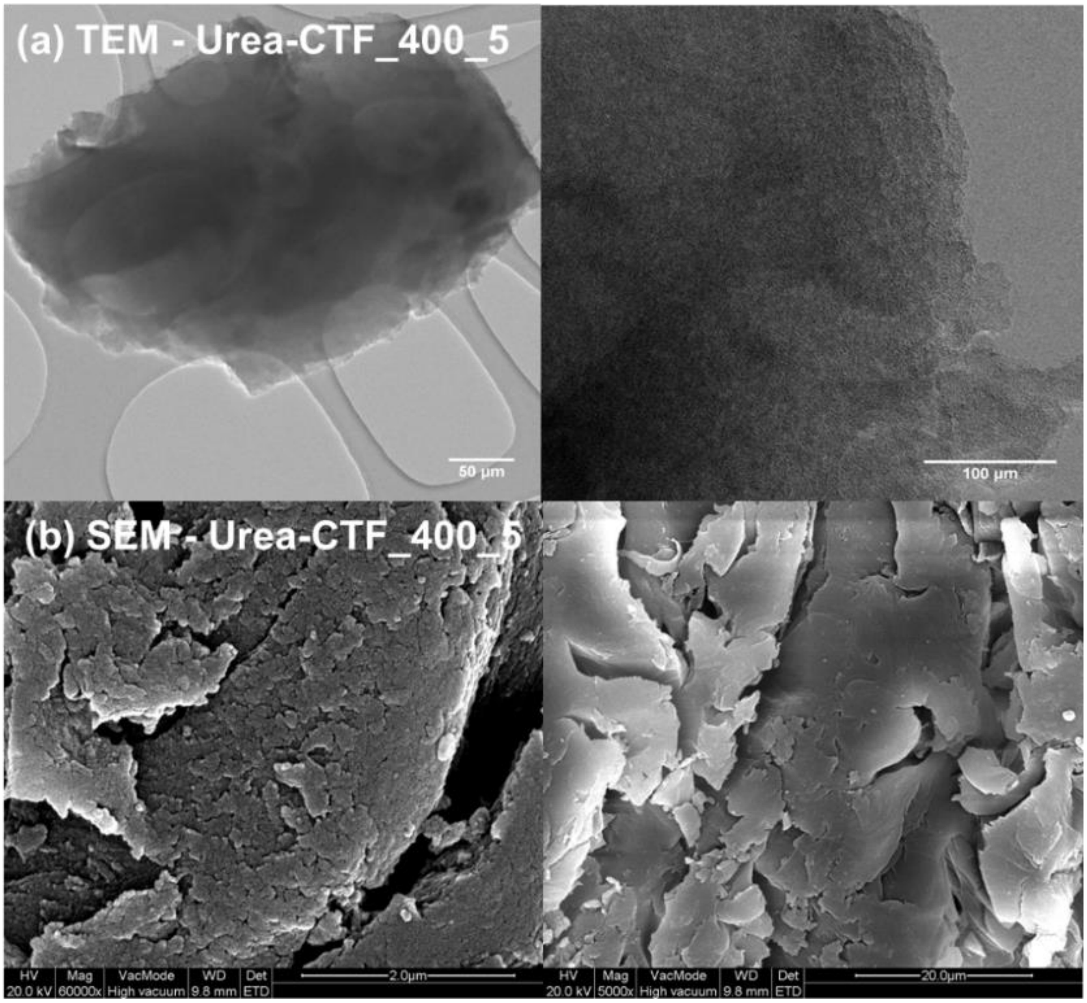

Figure S7: TEM and SEM images of Urea-CTF_400_5

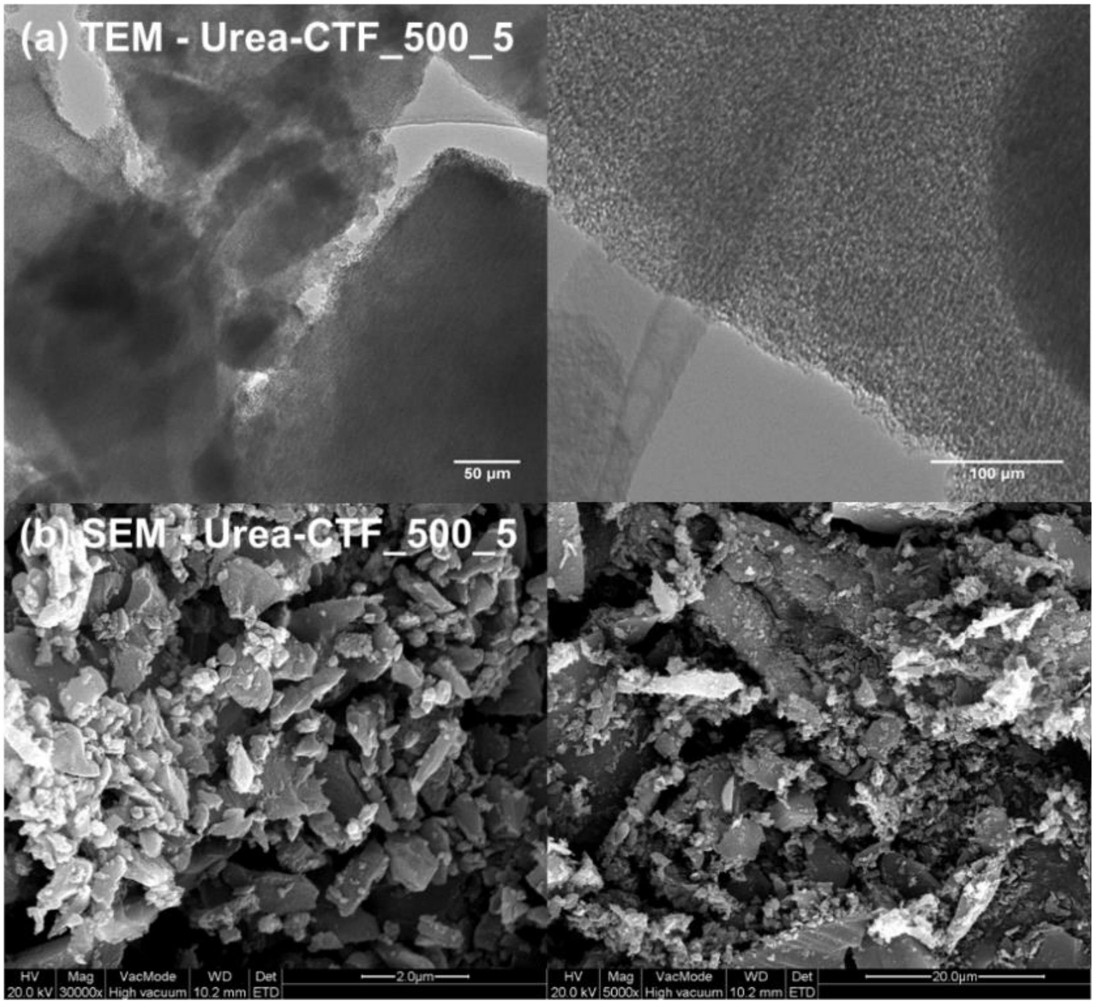

Figure S8: TEM and SEM images of Urea-CTF_500_5

Table S2: Gas uptake values for the Urea-CTFs at 1 bar pressure.

Material	C_2H_2 (mmol/g)		C_2H_4 (mmol/g)		CO_2 (mmol/g)		CH_4 (mmol/g)		N_2 (mmol/g)	
	273K	298K	273K	298K	273K	298K	273K	298K	273K	298K
Urea-CTF_400_5	3.9	2.8	2.9	2.1	2.8	1.8	0.88	0.56	0.22	0.15
Urea-CTF_500_5	3.8	2.6	2.9	1.9	3.1	1.5	0.69	0.43	0.21	0.13

Table S3: Isosteric heat of adsorption (Q_{st}) values for the Urea-CTFs.

Material	Q_{st} C_2H_2 (kJ/mol)	Q_{st} C_2H_4 (kJ/mol)	Q_{st} CO_2 (kJ/mol)	Q_{st} CH_4 (kJ/mol)
Urea-CTF_400_5	35.51	32.39	30.05	22.02
Urea-CTF_500_5	27.78	29.09	48.57	17.09

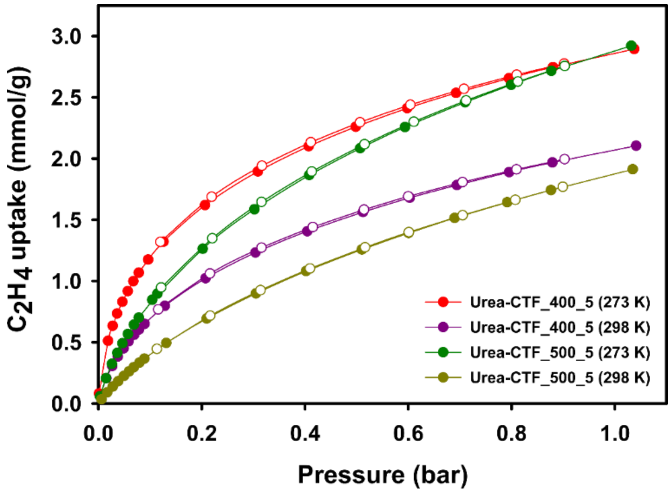

Figure S9: C_2H_4 uptake of Urea-CTF_400_5 and Urea-CTF_500_5.

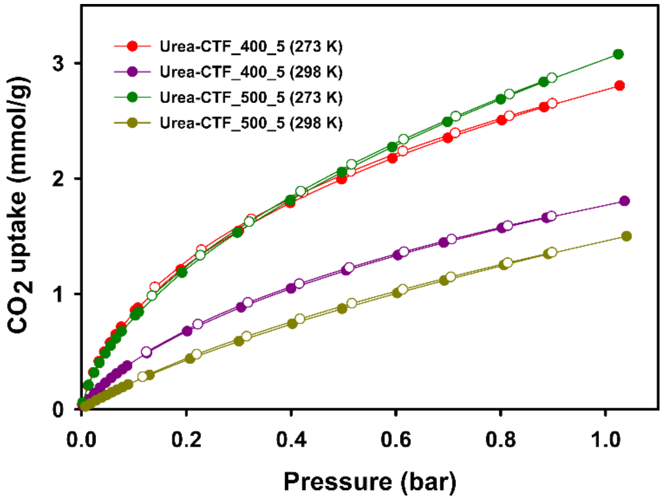

Figure S10: CO_2 uptake of Urea-CTF_400_5 and Urea-CTF_500_5.

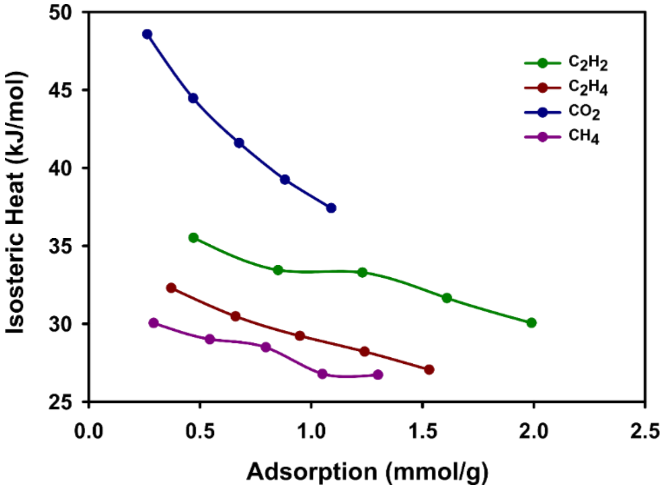

Figure S11: Isosteric heat of adsorption (C_2H_2, C_2H_4, CO_2, CH_4, N_2) for the Urea-CTF_400_5.

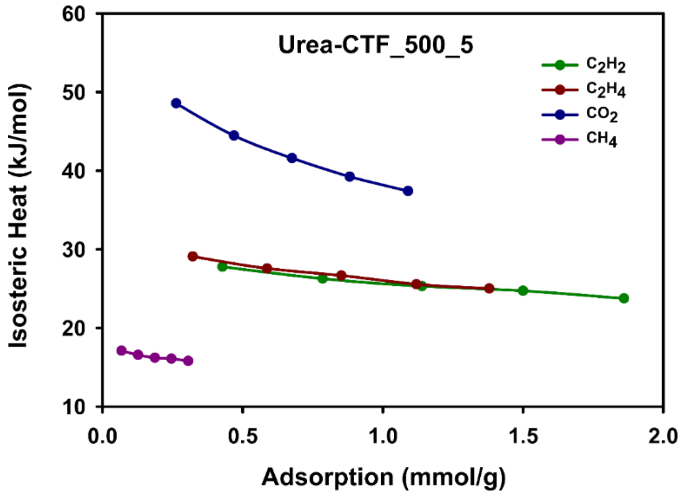

Figure S12: Isosteric heat of adsorption (C_2H_2, C_2H_4, CO_2, CH_4) for the Urea-CTF_500_5.

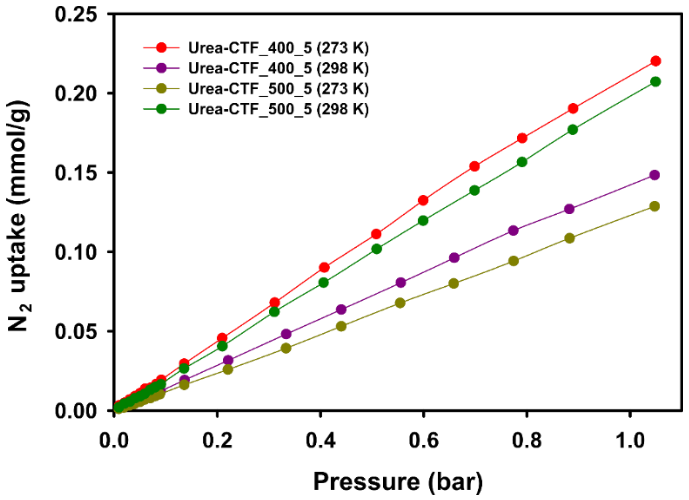

Figure S13: N_2 uptake of Urea-CTF_400_5 and Urea-CTF_500_5

Ideal Adsorbed Solution Theory (IAST)

The single component isotherm of C_2H_2, C_2H_4, CO_2, CH_4 and N_2, measured at 273K and 298K of the Urea-CTFs were fitted (Table S5) with the single-site Langmuir isotherm model using the equation,

$$q_{eq} = q_{max} \cdot \frac{K.p}{1 + K.p}$$

where, p is the pressure of the bulk gas at equilibrium with the adsorbed phase, q_{eq} is the adsorbed amount per mass of adsorbent, q_{max} is the maximum capacity of the site and K is the affinity coefficient of the site.

The adsorption selectivity was calculated using the following equation where qA=qC_2H_2, qC_2H_4 or qCO_2, and qB=qCH_4 or qN_2 represent the molar loadings of CTFs that is in equilibrium with a bulk fluid mixture with mole fractions of yA=0.5 and yB=0.5 for separation under 50/50=A/B conditions.

$$S = \frac{qA/qB}{yA/yB}$$

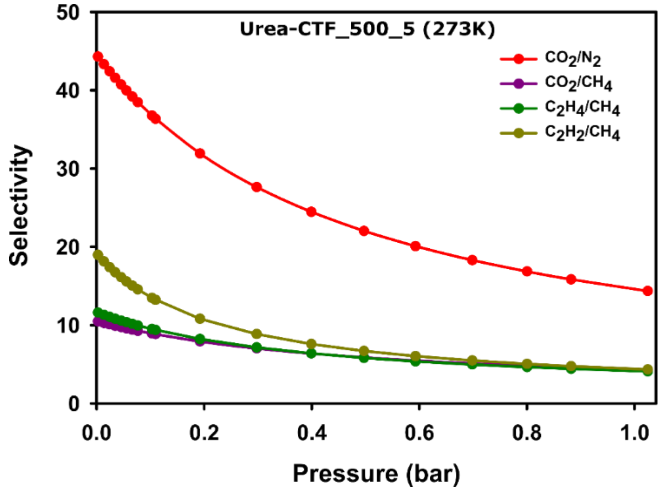

Figure S14: IAST selectivity of Urea-CTF_500_5 at 273K

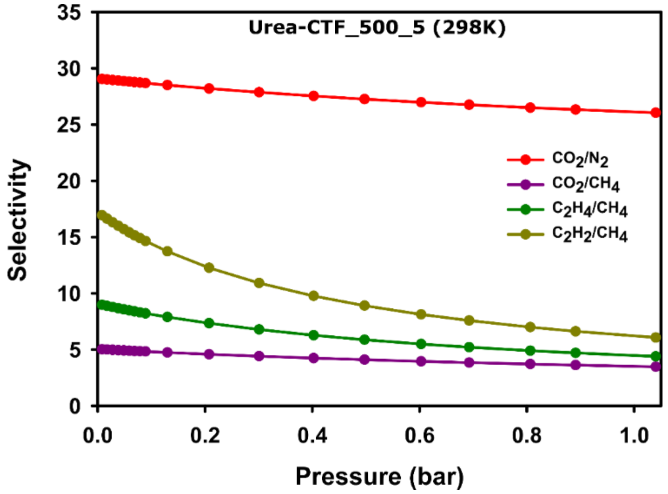

Figure S15: IAST selectivity of Urea-CTF_500_5 at 298K

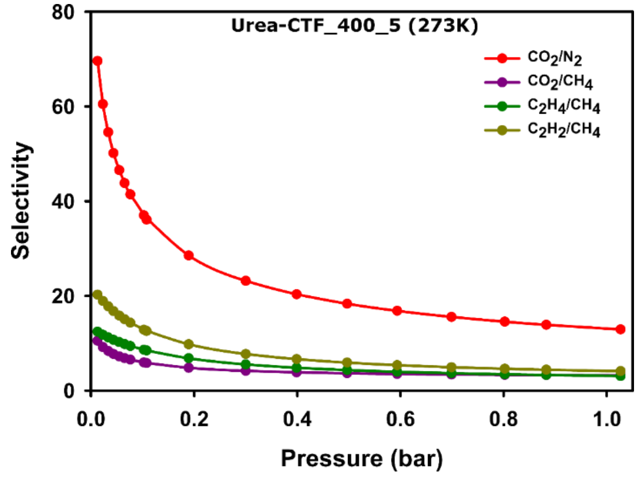

Figure S16: IAST selectivity of Urea-CTF_400_5 at 273K

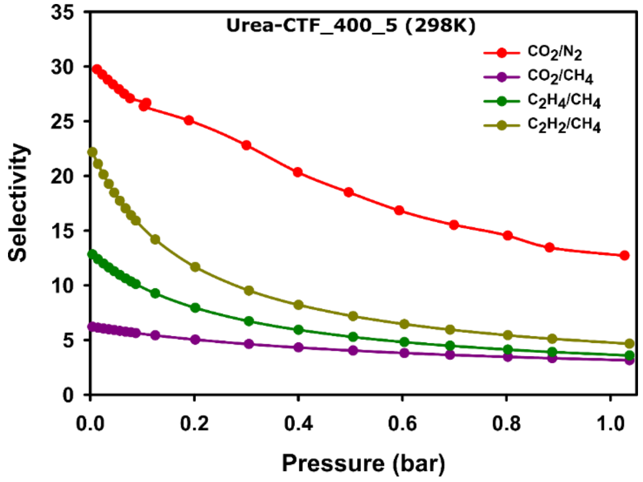

Figure S17: IAST selectivity of Urea-CTF_400_5 at 298K

Table S4: IAST selectivity values of the Urea-CTFs.

**Material**	**Selectivity C_2H_2/CH_4**		**Selectivity C_2H_4/CH_4**		**Selectivity CO_2/CH_4**		**Selectivity CO_2/N_2**	
	**273K**	**298K**	**273K**	**298K**	**273K**	**298K**	**273K**	**298K**
Urea-CTF_400_5	20.25	22.18	12.42	12.82	10.49	6.21	69.62	29.74
Urea-CTF_500_5	18.96	16.95	11.58	8.97	10.45	5.02	44.31	29.05

Table S5: Langmuir fit parameters for Urea-CTFs.

Material	C_2H_2			C_2H_4			CO_2			CH_4			N_2		
	q_{max}	K	R^2	q_{max}	K	R^2	q_{max}	K	R^2	q_{max}	K	R^2	q_{max}	K	R^2
Urea-CTF_400_5-273K	3.99	8.48	0.98	3.09	6.56	0.99	3.54	2.92	0.99	1.98	0.77	0.99	3.20	0.07	0.99
Urea-CTF_400_5-298K	3.04	5.28	0.98	2.50	3.69	0.99	2.80	1.58	0.99	2.22	0.32	1	6.43	0.023	0.99
Urea-CTF_500_5-273K	3.59	4.48	0.98	3.86	2.53	0.99	4.30	2.06	0.99	3.27	0.26	1	330.83	0.0006	0.99
Urea-CTF_500_5-298K	3.75	2.12	0.99	3.27	1.28	0.99	3.79	0.61	0.99	3.67	0.13	1	0.18	0.45	0.98

Gas separation simulations

For all the gas separation simulations, the 3Psim software (version 1.1.0.8) from the 3P instruments was used.

Initially, the adsorption isotherms (C_2H_2, C_2H_4, CO_2, CH_4) measured at 273K, 298K and 313K of Urea-CTF_400_5 are fitted using the Langmuir Adsorption Isotherm equation.

$$q_{eq} = q_{max} \cdot \frac{K.p}{1 + K.p}$$

Where q_{eq} = adsorption, q_{max}=maximum adsorption, K is the affinity constant, p=equilibrium pressure.

This fitting provides the affinity constant (1/bar), maximal loading (mmol/g), heterogeneity parameter and heat of adsorption (kJ/mol) for the corresponding temperatures.

Table S6: Fitting parameters of adsorption isotherms of Urea-CTF_400_5 at 298K

Gas	Affinity constant (1/bar)	Maximal loading (mmol/g)	R^2
C_2H_2	5.31	3.07	0.97
C_2H_4	3.73	2.51	0.99
CO_2	1.60	2.82	0.99
CH_4	0.33	2.19	0.99

*The marginal variation in these fitting parameters from previously calculated parameters for IAST selectivities is due to the inclusion of a third isotherm measured at 313K. Three isotherms are necessary to perform these simulations.

For determination of the adsorbent performance in mixed component systems at 298K, the affinity constant (1/bar), saturation capacity (mmol/g) and total pressure (1 bar) were used. Two plots each for C_2H_2 vs CH_4

(figure S19), C_2H_4 vs CH_4 (figure S20) and CO_2 vs CH_4 (figure S21) were simulated. In all cases, plot (i) – Gas composition constant, Pressure variable and plot (ii) – Gas composition variable, Pressure constant.

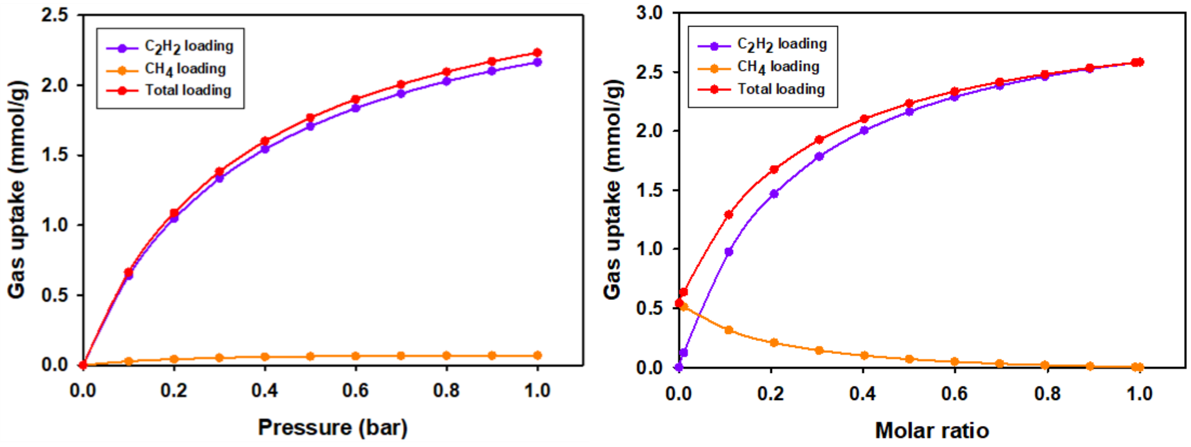

Figure S18: C_2H_2 vs CH_4 (mixed component simulation) at (left) constant gas composition and variable pressure and (right) variable gas composition and constant pressure

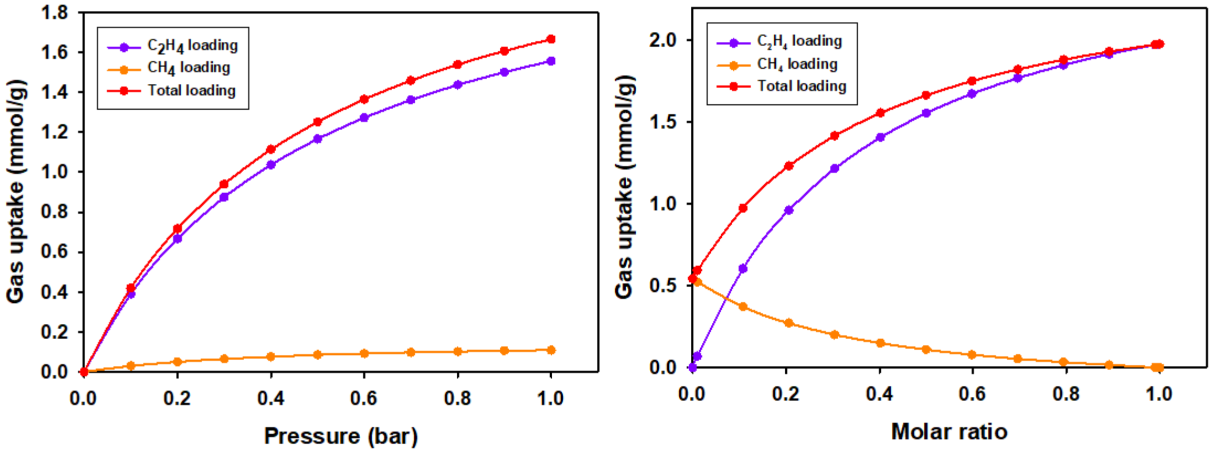

Figure S19: C_2H_4 vs CH_4 (mixed component simulation) at (left) constant gas composition and variable pressure and (right) variable gas composition and constant pressure

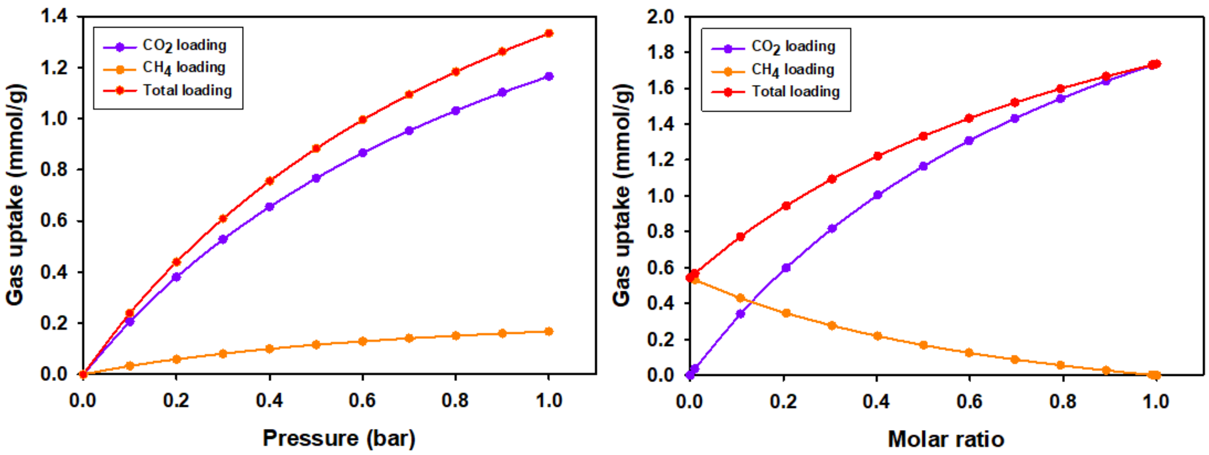

Figure S20: CO_2 vs CH_4 (mixed component simulation) at (left) constant gas composition and variable pressure and (right) variable gas composition and constant pressure

Finally breakthrough simulations were performed with defined height, diameter of the column, gas flow rate and mass of adsorbent at 298K and 1 bar pressure (table S7). The breakthrough plots for C_2H_2/CH_4, C_2H_4/CH_4 and CO_2/CH_4 are shown in figure 1c, figure S22 and figure S23.

Table S7: Breakthrough simulation parameters for Urea-CTF_400_5 at 298K

**Parameters**	**C_2H_2 vs CH_4**	**C_2H_4 vs CH_4**	**CO_2 vs CH_4**
Column height	15 cm	15 cm	15 cm
Column diameter	1 cm	1 cm	1 cm
Gas flow	20 ml/min	20 ml/min	20 ml/min
Temperature	298K	298K	298K
Pressure	1 bar	1 bar	1 bar
Adsorbent mass	3.5 g	3.5 g	3.5 g
Particle diameter	0.5 mm	0.5 mm	0.5 mm
Bed porosity	0.3	0.3	0.3
Carrier gas	He	He	He

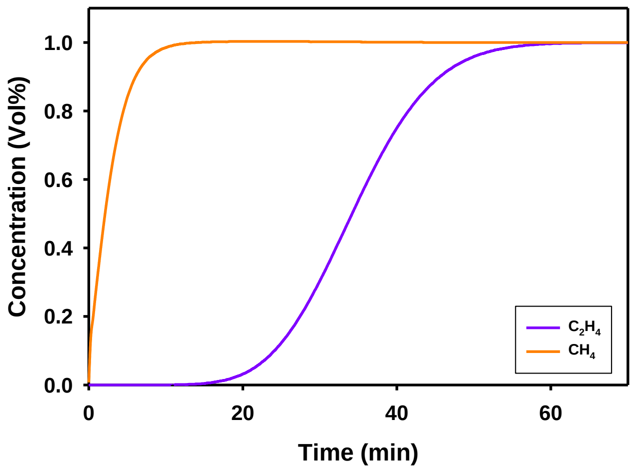

Figure S21: Breakthrough simulation of C_2H_4 vs CH_4 for Urea-CTF_400_5

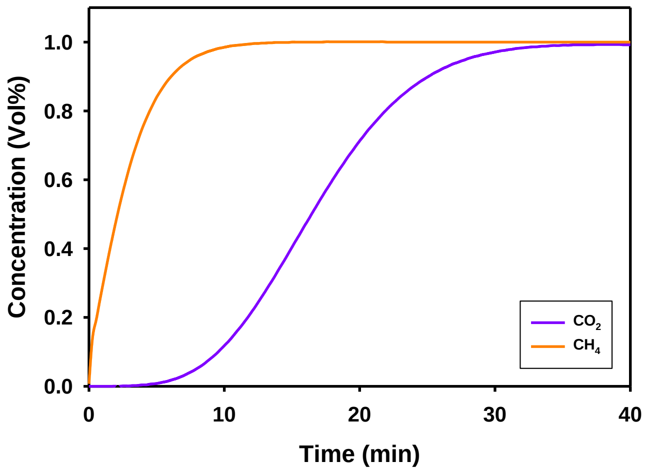

Figure S22: Breakthrough simulation of CO_2 vs CH_4 for Urea-CTF_400_5
